# Prevalence of viral hepatitis B and C infection and associated factors among pregnant women in southeast Ethiopia: community-based crossectional study

**DOI:** 10.3389/fgwh.2025.1508788

**Published:** 2025-02-04

**Authors:** Nuruzelam Mohammed, Jeylan Kassim, Ahmednur Adem Aliyi, Muhammed Jemal Abdurebi

**Affiliations:** ^1^ Public Health Department, School of Health Science, Goba Referral Hospital, Madda Walabu University, Robe, Ethiopia; ^2^ Public Health Department, Health Institute, Bule Hora University, Bule Hora, Ethiopia

**Keywords:** HBV, HCV, pregnancy, prevalence, associated factors

## Abstract

**Introduction:**

Hepatitis B or C infection during pregnancy increases the risk of vertical transmission, which is risky for the growing fetus and the newborn. In order to prevent such adverse effects and outcomes, it is crucial to understand the scope of the problem. However, absence of data on community-based Prevalence of viral hepatitis among pregnant women and conflicting evidence from facility-based study shows there is paucity of information on seroprevalence of Hepatitis B and C virus infection among pregnant women.

**Methods:**

A community-based cross-sectional study was conducted on 422 pregnant women selected from three selected kebeles of Robe town. Study participants were selected using systematic sampling technique. Data were collected through pretested interviewer administered questionnaire and three milliliter blood sample were collected and tested for HBsAgn and Anti-HCV Antibodies. Descriptive statistics such as frequency, mean, and standard deviation were used to summarize data. Binary logistic regression was used to identify factors associated with occurrence of Hepatitis B and C virus among pregnant women. Accordingly, variables with *P* value < 0.25 in bivariate logistic regression were declared as candidate for multivariable logistic regression. From multivariable logistic regression adjusted odds ratio with 95% confidence interval were computed. Those variable with *p*- value <0.05 were declared as factor associated with dependent variable.

**Results and discussion:**

A total of 410 pregnant women participated in the study, which yielded a response rate of 97.2%. The seroprevalence of hepatitis B and C virus infections was found to be 7.6%, and 2.2% respectively, whereas one (0.24%) woman was co-infected. History of dental extraction (AOR = 2.70, 95% CI 1.09, 6.69), hospital admission (AOR = 6.96, 95%CI 1.73, 27.99), household contact (AOR = 3.93, 95% CI 1.37, 11.25), tattooing (AOR = 3.50 95% CI 2.31, 12.35), sexually transmitted infection (AOR = 11.42 95% CI 3.10, 42.35) were significantly associated with HBsAg infection whereas history of blood transfusion (AOR 5.58, 95% CI 1.03, 30.05, *P* = 0.045) and household contact (AOR 7.49, 95% CI 1.34, 41.76) were significantly associated with HCV infection among pregnant women.

**Conclusions:**

The Seroprevalence of HBV and HCV was moderate endemicity according to WHO classification. Finding from present study shows different factors that plays great role in transmission of viral hepatitis.

## Introduction

Hepatitis is an infection of the liver caused by various infectious viruses and non-infectious agents that can lead to a range of health problems, some of which can be fatal. Types A, B, C, D, and E are the five main hepatitis virus strains that can cause both acute and long-term liver infections. Hepatitis B and C, in particular, are the most common cause of chronic liver disease and have the greatest public health significance (1). Despite the availability of vaccines and antiviral treatment, hepatitis B and hepatitis C viral infections remain public health problems because they cause long-term infection of the liver, leading to significant morbidity and mortality (2).

According to estimates from WHO, 296 million people have chronic hepatitis B infection in 2019, with 1.5 million new infections per year leading to 820,000 deaths, mostly from cirrhosis and hepatocellular carcinoma, with most of these deaths occurring in developing countries (3) while approximately 58 million (3%) of the world's population have chronic hepatitis C virus infection, leading to 1.5 million new infections per year leading to 290,000 deaths, mostly from cirrhosis and hepatocellular carcinoma, most of them in East Asia and sub-Saharan Africa (4). According to a report from WHO, the African region accounted for 26% of the global burden of hepatitis B and C and 125,000 related deaths in 2020 (5).

Different prevalence rates have been reported among pregnant women in the African continent; for instance, the prevalence of HBV infection among pregnant women in sub-Saharan Africa ranges between 2.4% in Ethiopia (6) and 11.8% in Uganda (7). Prevalence of HCV among pregnant women in sub-Saharan Africa was estimated to be 3% (1). Ethiopia is in the region where Hepatitis B infection prevalence is labelled hyperendemic with a rate between 8 and 12%, whereas Hepatitis C prevalence is estimated at not less than 2.5% (8). In Ethiopia, a metanalysis from few available facility based crossectional studies report fa national pooled prevalenceof HBV and HCV among pregnant women to be 7.4% and 3.14%, respectively (9). Viral Hepatitis in pregnancy is closely linked to a high risk of maternal complications and bad birth outcomes. In India, during 2020 it resulted in 19.2% of maternal mortality and 42.6% of fetal death among those infected (3).

Vertical transmission of the hepatitis C virus from mother to child occurs in 3%–10% of pregnancies complicated by maternal HCV infection and is the most common cause of pediatric chronic HCV infection (10) that appears to be linked to the level of viremia in the pregnant woman rather than the route of infection (11). Unsafe healthcare procedures and injection drug use were the leading causes of HCV infection, accounting for 1.75 million new infections in 2015. HBV causes hepatitis of altering severity and remains in 95% of children and 10% of adult patients; vertical transmission plays a great role (12). Prevention of mother-to-child transmission of HBV is fundamental for reducing the burden of the disease in sub-Saharan Africa, where it is endemic, by implementing maternal screening combined with post-exposure prophylaxis, provision of first HBV vaccination immediately after delivery in all infants of HBsAg-positive mothers, together with immunoglobulin prophylaxis are effective methods (13).

In Ethiopia since 2007, efforts have been made to prevent and control viral hepatitis by integrating the B vaccine into the existing EPI program for children. In addition, a five-year (2016–2021) national strategic plan for the prevention and control of viral hepatitis was developed to reduce morbidity and mortality attributable to viral hepatitis by promoting prevention; however, the response to viral hepatitis was fragmented and non-existent in all public health facilities. In particular, routine screening of pregnant women for viral hepatitis interrupted infection in many public health facilities due to a lack of diagnostic equipment, which was due to financial constraints (8). There has been a paucity of nationwide studies measuring the burden of viral hepatitis infection in different socioeconomic, geographic, and demographic subgroups in the last three decades, and the studies available in Ethiopia have been limited and outdated. Data are only available from facility-based studies, and meta-analyses are limited to a subpopulation within a few health facilities. The lack of adequate and up-to-date data on the prevalence of viral hepatitis in pregnant women and the inconsistent results from previous facility-based studies indicate that there is limited information on the extent of the problem at the community level. Conducting a community-based prevalence study among pregnant women was, therefore, crucial and has endless benefits.

The aim of the present study was therefore to determine the prevalence of HBV and HCV infections and associated factors among pregnant women at the community level in Robe town administration southeast, Ethiopia.

## Methods and materials

### Study area, design, and period

The community-based crossectional study was conducted among pregnant women in three selected villages (kebeles) of Robe Town Administration, Oromia Region, southeast of Ethiopia, from March 1 to May 30, 2022.

### Population

The study was conducted among pregnant women at the community level to assess the prevalence of HBV and HCV infection. All selected pregnant women in the study area were included in the study, while those who refused to provide written consent were excluded.

Informed written consent was obtained from each participant after a clear explanation of the objective, their right to decide on participation, risks and benefits of the study. Those who couldn't read and write gave their consent by providing a finger signature after a clear oral explanation.

### Sample size determination and sampling methods

The sample size was calculated by using a single population proportion formula for sample size calculation by taking a proportion of 50%, 95% confidence level, 5%margin of error, and 10% non-response rate. This gives the final sample size of 422. Accordingly, the study was conducted among 422 pregnant women from March 1 to May 30, 2022. To reach the final participants, the first three out of six kebeles in the Robe town administration were randomly selected by using their names as the sampling frame. The total sample size was proportionally allocated to each selected kebeles based on their total population size, and then a systematic sampling technique was used to select a total of 422 pregnant women from selected kebeles. Accordingly, the first woman was selected by using lottery methods, while the rest were systematically selected with two skip intervals.

Therefore, every second pregnant woman who was registered and gave consent in the selected kebeles was included in the study until the calculated sample size was reached within three months of data collection.

### Variables and measurements

#### Dependent Variable

Serostatus of hepatitis C virus in pregnant women and serostatus of hepatitis B virus in pregnant women.

#### Independent variables

Sociodemographic characteristics such as maternal age, marital status, education level, family income, occupation.

Obstetric history: such as gravidity, parity, abortion, place of delivery.

Healthcare-related characteristics such as a history of Hospital admission, history of blood transfusion, contact with family members with liver disease, history of surgical procedures and dental procedures.

Risky behaviours and cultural practices such as tattooing, nose piercing, ear piercing, genital mutilation, sharing of sharp objects, and polygamous marriages.

Sexual history, including multiple sexual partners and sexually transmitted infections.

### Operational definitions

Hepatitis B surface antigen (HBsAg):- Hepatitis B surface antigen (HBsAg): A marker present in individuals who are currently infected with HBsAg (i.e., individuals with acute and chronic infection (14).

Seropositive: is the status of a person who reacts positively to a serological test.

Sero-negative:- the status of a person who reacts negatively to a serological test (14).

Anti–HCV antibodies: Anti-HCV antibodies usually develop 2–6 months after exposure during the acute phase of infection and persist throughout life. Accordingly, anti-HCV has been described as positive for active HCV infection (acute or chronic) and cured from previous infection while negative for no active HCV infection (15).

### Data collection and quality control

Data were collected by a trained data collector using a pre-tested, interviewer-administered questionnaire adapted from similar studies (6, 16–18). Data on sociodemographic characteristics, sociocultural risk behaviours and practices, and health-related factors were collected in a face-to-face interview by two midwives recruited as data collectors. Two experienced laboratory professionals collected five ml of venous blood from a peripheral vein in plain tubes from all eligible pregnant women under aseptic conditions and transported them to the laboratory. Standard operating procedures were strictly followed at each stage of the laboratory analysis. The blood samples were centrifuged for at least 15 min at room temperature at 3,000 rpm (RPM). Excellent quality According to the instructions of the manufacturer Bio Panda, a one-step HBsAg test strip was used to detect hepatitis B surface antigen (HBsAg), while the Best one-stage HCV test strip was used to detect antibodies against HCV.

The HBV test had a sensitivity and specificity of 98.89 and 98.87 percent, respectively, while for the anti-HCV test, they were 93.3 and 99.5%, respectively. The presence of a red band indicates a positive result, while its absence indicates a negative result. The results of the rapid test were communicated to the study participants and made available as soon as possible.

To ensure the quality of the data, the data collectors and the supervisor were trained before the actual data collection started. A pilot-test was also conducted on 5% of the study sample which are not included in the actual study (21 samples), and amendments were made based on the results of the pre-test. Supervisors and investigators checked the collected data daily for completeness and consistency. Completed questionnaires were assigned a code, and data entry was double-checked. The quality of the serological data was kept by the application of the laboratory's standard operating procedures for sample collection, storage and analysis of blood samples.

### Ethical consideration

The study was approved by the Madda Walabu University Research and Development Directorate. Written informed consent was obtained from each study participant before data collection.

The results were communicated to the participants with appropriate counselling (disease progression, screening of partners and sero-vaccination of newborns). All infected pregnant women were counselled on the disease and referred for proper specialized care. For this purpose, the authors have access to information about the participants during the study.

### Data processing and analysis

The collected data were reviewed, coded and entered into Epidata version 4.6 and analyzed by using SPSS version 26. Descriptive statistics of the different variables were obtained, and the results were presented in text, graphs, and frequency tables. Bivariate logistic regression was performed to identify factors associated with the occurrence of hepatitis B and C virus infection. Accordingly, variables with a *P*-value < 0.25 were declared candidates for multivariable logistic regression in multivariable logistic regression. The adjusted odds ratio with 95% confidence intervals was calculated from the multivariable logistic regression. The variables with a *p*-value < 0.05 were declared a factor associated with the dependent variable. The Hosmer-Lemeshow goodness-of-fit test was used to assess model adequacy.

## Results

### Socio-demographic characteristics of study participants

For the present study, 422 pregnant women were recruited, of whom 410 responded completely, representing a response rate of 97.2%. The mean age of the study participants was 25.53 with standard deviation of 5.86 (range 16–44) years; 160 (39.0%) were residents of Café Donsa Kebele, 301 (73.4%) were of Oromo ethnicity, and most of them, 218 (53.2%), attended primary school. In addition, 388 (94.6%) were married and 308 (75.1%) were housewife in occupation. As illustrated in Table 1.

**Table 1:** Socio-demographic characteristics of study participant.

Parameter	Frequency (*n* = 410)	Percent (%)
Age category in years		
15–19	58	14.1
20–29	250	61.0
30–39	90	22.0
40–49	12	2.9
Residence		
Baha Biftu	106	25.9
Oda Robe	144	35.1
Cafe Donsa	160	39.0
Ethnic group		
Oromo	301	73.4
Amhara	88	21.4
Somali	5	1.2
Others	16	3.9
Educational level		
Illiterates	72	17.6
Primary (1–8)	218	53.2
Secondary (9–12)	87	21.2
Above grade 12	33	8.0
Marital status		
Cohabited	14	3.4
Married	388	94.6
Divorce	8	2.0
Occupation		
Government employee	26	6.3
Private employee	76	18.5
House wife	308	75.1
Wealth index		
Poorest	83	20.2
Poor	89	21.7
Medium	74	18
Rich	82	20
Richest	82	20

### Prevalence of hepatitis B and C virus infection among study participants

The Overall prevalence of HBsAg and anti-HCV antibodies was 31 (7.6%) with 95 CI: 5.1%–10.2% and 9 (2.2%)% CI: 1%–3.7%, respectively, whereas 1 (0.24% with 95% CI, 0.01, 1.19)) was co-infected among all study participants. As shown in Table 2.

**Table 2:** Seroprevalence of hepatitis B and C virus infection among study participants.

Outcome variable status	Frequency (*n* = 410)	Total (%)
HBsAg		
Negative	379	92.4
Positive	31	7.6 (5.1%–10.2%)
Anti -HCV antibodies		
Negative	401	97.8
Positive	9	2.2 (1–3.7)
Co-infection (HBsAg and HCV)		
Negative	409	99.76
Positive	1	0.24 (0.01, 1.19)
Overall serological prevalence of viral hepatitis		
Negative	370	90.25
Positive	40	9.75 (7.14, 13.15)

### Bivariate and multivariable logistic regression analysis for factors associated with the prevalence of hepatitis HBV among pregnant women

In this study, dental extraction, hospital admission, history of contact, history of tattoos, and history of sexually transmitted infection were found to be significantly associated with the presence of HBsAg infection (*P* < 0.05).

Accordingly, pregnant women who had a history of tooth extraction have 2.7 (AOR = 2.70, 95% CI 1.09, 6.69, *p* = 0.032), those with a history of hospital admission have 6.96 (AOR = 6.96, 95% CI 1.73, 27.99, *P* = 0.006), those with a history of household contact have 3.93 (AOR = 3.93, 95% CI 1.37, 11.25, *P* = 0.011), having a history of tattoos have 3.5 (AOR = 3.50 95% CI 2.31, 12.35, *P* = 0.021) and history of sexually transmitted infection have 11.42 (AOR = 11.42 95% CI 3.10, 42.35, *P* = 0.001) more likelihood of contracting hepatitis B virus than their counterpart respectively. As shown in Table 3.

**Table 3:** Bivariate and multivariable logistic regression analysis for factors associated with prevalence of hepatitis B virus among pregnant women.

Variables response	HBV status		COR (95%CI)	*P*-value	AOR (95%CI)	*P*-value
	Negative *n* (%)	Positive *n* (%)				
Parital status						
Primipara	93 (22.7)	5 (1.2)	1	0.414	1	0.414
Para two	99 (24.1)	7 (1.7)	1.31 (0.40, 4.29)	0.650	0.893 (0.174,4.57)	0.892
Multipara	187 (45.6)	19 (4.6)	1.89 (0.68, 5.22)	0.220	0.95 (0.221,4.073)	0.944
Dental extraction						
No	275 (67.1)	11 (2.7)	1	0.001	1	0.032**
Yes	104 (25.4)	20 (4.9)	4.808 (2.22,10.37)		2.70 (1.09, 6.69)	
Hospital admission						
No	261 (63.7)	4 (1)	1	0.001	1	0.006**
Yes	118 (28.8)	27 (6.6)	14.93 (5.10,43.62)		6.96 (1.73, 27.99)	
History of contact						
No	355 (86.6)	21 (5.1)	1	0.001	1	0.011**
Yes	24 (5.9)	10 (2.4)	7.04 (2.98, 16.62)		3.93 (1.37,11.25)	
Abortion						
No	306 (74.6)	17 (4.1)	1	0.001	1	0.312
Yes	73 (17.8)	14 (3.4)	0.29 (0.13, 0.61)		1.63 (0.63, 4.21)	
Tattooing						
No	348 (84.9)	21 (5.1)	1	0.001	1	0.021**
Yes	31 (7.6)	10 (2.4)	5.34 (2.31, 12.35)		3.50 (2.31, 12.35)	
History of STI						
No	361 (88)	22 (5.4)	1	0.001	1	0.001**
Yes	18 (4.4)	9 (2.2)	8.2 (3.30, 20.35)		11.42 (3.10,42.35)	
Multiple sexual partners						
No	351 (85.6)	24 (5.9)	1	0.06	1	0.180
Yes	28 (6.8)	7 (1.7)	3.65 (1.45,9.22		2.37 (0.67, 8.40)	

COR, crude odd ratio; AOR, adjusted odd Ratio; 1, reference.

** Statistically significant on multivariable logistic regression analysis at *p*-value < 0.05.

### Bivariate and multivariable logistic regression analysis for factors associated with the prevalence of hepatitis C virus among pregnant women

In the final model of multivariable logistic regression, two variables remain as factors significantly associated with the occurrence of hepatitis C virus among pregnant women at *p*-value <0.05. These were having a history of blood transfusion and a history of household contact with a family member having liver disease.

Pregnant women who had previously received blood transfusions had 5.58 times higher odds ratio than their counterparts to getting HCV infection (AOR 5.58 95% CI 1.03, 30.05, *P* = 0.045), and those having a history of household contact with a family member having liver disease have 7.49 more likelihood than those without a history of household contact to contract HCV infection (AOR 7.49 95% CI 1.34, 41.76, *P* = 0.022). Out of the variables identified to be associated with HCV infection in the bivariate analysis, dental extraction, history of husband polygamy, history of abortion, history of tattoos, and history of nose piercing were not found to be significantly associated with HCV Infection in multivariable logistic regression analysis. As illustrated in Table 4.

**Table 4:** Bivariate and multivariable logistic regression analysis for factors associated with the prevalence of hepatitis C virus among pregnant women in Robe town 2022.

Variables category	Freq *n* (%)	HCV test result		COR (95% CI)	*P*-Value	AOR (95% CI)	*P*-value
		Negative *n* (%)	Positive *n* (%)				
Dental extraction							
No	186 (69.6)	283 (69)	3 (0.7)	1		1	
Yes	124 (30.2)	118 (28.8)	6 (1.5)	4.79 (1.18,19.49)	0.028	2.97 (0.63,14)	0.657
Hospital admission							
No	265 (64.6)	263 (64.1)	2 (0.5)	1		1	
Yes	145 (35.4)	138 (33.7)	7 (1.7)	16.67 (1.37,32.54)	0.019	2.38 (0.32,17.49)	0.392
Blood transfusion							
No	357 (87.1)	353 (86.1)	4 (1)	1		1	
Yes	53 (12.9)	48 (11.7)	5 (1.2)	9.19 (2.38, 35.42)	0.001	5.58 (1.03,30.05)	0.045**
History of contact							
No	376 (91.7)	371 (90.5)	5 (1.2)	1		1	
Yes	34 (8.3)	30 (7.3)	4 (1)	9.89 (2.52,38.79)	0.001	7.49 (1.34,41.76)	0.022
Abortion							
No	324 (79)	320 (78)	4 (1)	1		1	
Yes	86 (21)	81 (19.8)	5 (1.2)	4.93 (1.29, 18.80)	0.019	2.33 (0.47, 11.41)	0.293
Tattooing							
No	369 (90)	363 (88.5)	6 (1.5)	1		1	
Yes	41 (10)	38 (9.3)	3 (0.7)	4.77 (1.14, 19.87)	0.032	4.7 (0.73, 30.30)	0.103
Husband polygamy							
No	350 (85.4)	345 (84.1)	5 (1.2)	1		1	
Yes	60 (14.6)	56 (13.7)	4 (1)	4.92 (1.28, 18.91)	0.020	4.38 (0.78,24.47)	0.092
Multiple sexual partners							
No	375 (91.5)	368 (89.8)	7 (1.7)	1		1	
Yes	35 (8.5)	33 (8)	2 (0.5)	3.18 (0.63,15.96)	0.159	1.36 (0.15,11.77)	0.779

COR, crude odd ratio; AOR, adjusted odd ratio.

** Statistically significant on multivariable logistic regression analysis at *p*-value < 0.05., 1 = reference.

## Discussion

Hepatitis B and C virus infections (HBV, HCV) are serious health concerns that affect people all over the world. The most common cause of hepatic impairment in pregnancy is viral hepatitis. According to WHO definitions, there are three levels of endemicity for HBV infection: low endemicity (less than 2% seropositive), intermediate endemicity (2% to 7% seropositive), and high endemicity (>8% seropositive), whereas HCV prevalence is classified as high (>3.5%), Intermediate (1.5% - 3.5%), and low (1.5%) (1). In the present study, the prevalence of Hepatitis B and Hepatitis C infections among pregnant women was 7.6% (95 CI: 5.1–10.2) and 2.2% (95% CI: 1–3.7), respectively, whereas 0.24% (95 CI, 0.01, 1.19)) were co-infected among all participating pregnant women.

Prevalence of Hepatitis B and C in the present study was consistent with findings from various studies conducted around the world (19–22). These findings were not in line with the results from different studies conducted previously (12, 21–30). The reasons for variations of prevalence HBsAg and Hepatitis C antibodies in Ethiopia and elsewhere might be due to differences in geographical areas, level of awareness on a different route of viral transmission, sociodemographic differences, cultural practices, and behavioural practices toward the risk of HBV and HCV infection. Thus, the relative increase in the prevalence of HBsAg and HCV antibodies observed in the present study among pregnant women suggests that the present study area is one of the priority target areas for the prevention and control of hepatitis in the country. The high occurrences of these viruses could be due to low awareness and preventive practice in the community. Relatively poor consideration from policy makers could also contribute for the occurrences of these problems.

In the present study, a history of tooth extraction is positively related with likelihood of contracting HBV infection than those who don't have a history of tooth extraction (AOR = 2.70, 95% CI 1.09, 6.69, *p* = 0.032). This finding was consistent with study reports from Southwest Ethiopia (31) and Northwest Ethiopia (30) showing a significant association among study participants having a history of dental extraction with HBsAg infection. However, it conflicted with the study finding report from Saudi Arabia in which dental extraction was not significantly associated with HBV infection (32). The similarity in results could be attributed to comparable healthcare practices and potential gaps in infection control during dental procedures in these regions. In contrast, the study from Saudi Arabia found no significant association, which may be due to stricter sterilization protocols and better healthcare infrastructure. Differences in awareness levels, access to healthcare services, and sociodemographic profiles of study participants may further explain these discrepancies. Additionally, cultural attitudes toward seeking dental care and the frequency of informal or unregulated dental practices might contribute to the variations observed.

History of hospital admission among pregnant women was positively and significantly associated with likelihood of having HBV infection (AOR = 6.96, 95% CI 1.73, 27.99, *P* = 0.006). The present study finding was in line with a study from a different region indicating that pregnant women with a history of hospitalization were 97 times more likely than pregnant women without a history of hospitalization to be reactive to HBsAg infection (AOR = 0.030, 0.002–0.377) (33) and study report from Saudi Arabia found that pregnant women who had previously been hospitalized had a substantial correlation with HBV positivity (AOR = 2.2,0.96–5.43, *P* = 0.05) (32).

These indicate that hospitalization may expose individuals to unsafe medical practices or blood products. Variations in healthcare standards, such as the availability of single-use medical supplies and adherence to infection control measures, likely influence these outcomes. Higher exposure to improperly sterilized equipment or unsafe transfusions could exacerbate the risk in regions with limited resources. The findings in the present study found that a previous history of household contact with a family member having the liver disease were related with more chance of contracting HBsAg infection (AOR = 3.93, 95% CI 1.37, 11.25, *P* = 0.011). The finding in the present study was incongruent with the previous study from Sudan (34) Turkey (Antioch, University Hospital) (16), Felege Referral Hospital (30), the survey reported from Ethiopian hospitals (35) reporting significant association of having the previous history of household contact with a family member having liver disease and HBsAg infection. However, the findings from this study conflicted with other study reports, indicating there was no association between the previous history of contact and HBsAg infection (36).

It might be due to study participants’ lack of awareness of the transmission mode of the hepatitis virus, less precaution of sharing sharp material, traditional practices, and unsafe sexual practices. Furthermore, the possible explanation might be having contact with someone who is chronically ill or a carrier of hepatitis B virus may increase the probability of exposure to the source of infection, suggesting household/close contact to be a potential risk factor for viral hepatitis transmission in the present study which demands great attention in providing health education for the community regarding mode of viral hepatitis transmission and implementation of preventive measure to be taken while providing care for a family member having liver disease. The other possible reasons for the inconsistency with other reports might stem from differences in cultural norms related to caregiving practices and the extent of close contact with infected family members.

A history of Tattoos is related positively with having HBV infection among pregnant women (AOR = 3.50 95% CI 2.31, 12.35, *P* = 0.021). The finding in the present study was consistent with study findings from the Ghana Ashanti Region, in which tattooing was found to be significantly associated with HBV infection (28). In contrast to the present study finding report from a study conducted in Nigeria to assess the risk of HBV infection during pregnancy, it was found that the presence of tattoo or scarification marks did not differ between the two groups (HBsAg reactive or non-reactive), (*p* > 0.05) (23). These might be due to the low level of community awareness of infection prevention and transmission mode of viral hepatitis. Observed differences might be due to variations in the sample size of study participants, awareness of the transmission mode of hepatitis viruses, safety precautions, traditional practices, and the culture of the society. Also, the variation may reflect differences in tattooing practices, including the use of unsterilized equipment, and the regulatory environment surrounding body art procedures. Additionally, differences in community awareness about infection risks associated with tattooing may play a role. The implication of the present study suggests the need for great attention to be given to community awareness creation to avoid risky socio-cultural behaviours that may contribute to the transmission of viral hepatitis.

History of sexually transmitted disease were highly related with infection with HBV among the study participants (AOR = 11.42 95% CI 3.10, 42.35, *P* = 0.001). The finding in the present study was in line with the study finding from Harar town (37) suggesting pregnant mothers who experienced a previous history of sexually transmitted infection were ten times more likely to be infected by HBV when compared to their Counterparts. This is because a history of sexually transmitted infection is closely related to involvement in heterosexual practice or having multiple sexual partners and unprotected sexual intercourse. Alternatively, it suggests that STI history is a strong marker for unsafe practices contributing to HBV spread. Variations across regions may relate to differences in sexual health education, availability of protective measures, and healthcare access for STI management. Hence, the present study finding suggests having a history of sexually transmitted infections is a risk factor for HBV infection and recommends the need for prevention of transmission of HBV infection primarily starts with behavioural change on practising safer sex.

Regarding factors associated with seropositivity of the hepatitis C virus, having a history of blood transfusion and household contact with a family member having the liver disease were significantly associated with HCV infection among pregnant women at (*p*-value < 0.05). The participant who had previously received blood transfusions were six times more likely than their counterpart to get HCV infection (AOR 5.58 95% CI 1.03, 30.05, *P* = 0.045).

The findings from the present study were consistent with study reports from Ghana (28) and Pakistan (38, 39) reporting that blood transfusion has been recognized as a risk factor for the acquisition of HCV infection. In contrast to the present study, a study from Nigeria found that blood transfusion had no significant association with contracting HCV infection (17). The reason for similarity might be due to high-risk populations that are more heavily exposed to blood products, suggesting that this effect may be due to ongoing and repeated exposures to HCV infection through the medical care system. The source of variation might be due to failure to adequately screen donor's blood because of financial limitations highlighting the need to strengthen Universal screening of blood and blood products for the prevention of transfusion-transmissible diseases, including hepatitis C virus.

History of household contact with a family member having the liver disease among pregnant women is positive risk factors for the occurrence of HCV infection (AOR 7.49, 95% CI 1.34, 41.76, *P* = 0.022). In contrast to the present study, study reports from Bahirdar (18) and Sudan (34, 40) found that none of the expected risk factors (history of blood transfusion, surgery, dental manipulations, tattooing circumcision, etc., and other sociodemographic factors) associated for seropositivity of HCV had been identified. The source of observed discrepancy might be due to differences in socio-cultural behaviours, sample size, and efficiency of the method used in screening anti-HCV antibodies. In addition, discrepancies may arise from differences in awareness, hygiene practices, or the efficiency of HCV screening methods. Household contact in some settings might involve closer interpersonal interactions or shared use of sharp objects, which are potential transmission vectors.

Socio-demographic variables like age, residence, wealth index, marital status and educational level, occupation, and parity, as well as risk factors such as the previous history of abortion, tattoos, multiple sexual partners, and previous history of hospital admission, were not significantly associated with HCV infection at (*P* > 0.05). Considering the magnitude and severity of viral hepatitis, findings from the present indicate prevention and control of viral hepatitis needs a high degree of attention by all stakeholders, government, and funding agencies.

### Strength and limitation

The study was conducted at the community level to address all pregnant women in the study area. Data were collected by experienced and trained data collectors. Standard operating procedures were followed throughout blood sample collection and tests. However, the study was not without limitations.

However, this study has some limitations. The present study used HBsAg and Anti-HCV Seromarker for the detection of HBV and HCV infection. The screening test used for the detection of Hepatitis C has relatively lower sensitivity. Other sero-markers other than HBsAg and Anti-HCV Antibodies like Hepatitis B core antibodies (Anti-HBc), which would have helped to identify acute infection and determine viral load, were not possible to use in this study due to logistical issues. We did not perform high-resolution abdominal ultrasound, and we also didn't perform serial liver enzyme tests to determine the activity of infection. Also, HCV PCR was not utilized to differentiate acute Hepatitis C infection from chronic one. However, we referred every positive mother to nearby Hospitals for further physician investigation and management. Therefore, large-scale community-based study design using the molecular technique is needed in the future.

## Conclusion

The prevalence of HBsAg and Anti HCV Antibodies was 7.6% and 2.2% respectively. Prevalence of Co-infection with both HBV and HCV was 0.24% among all study participants. This indicates intermediate endemicity among participating pregnant women according to WHO classification (41). A history of dental extraction, hospital admission, contact with family members who had liver diseases, history of tattooing, and history of sexually transmitted diseases was significantly associated with the occurrence of the Hepatitis B virus among pregnant women. A history of blood transfusion and contact with liver disease patients were significantly associated with the Hepatitis C virus among pregnant women.

Therefore, there is a need to institute public health measures to reduce viral hepatitis transmission, including avoiding socio-cultural malpractice and creating awareness in the community about transmission and prevention methods, in addition to ensuring proper implementation of universal precautions at the community level.

Because of the possible vertical transmission of infection from mother to child we also recommend early provision of HBV vaccine for newborns immediately to prevent possible hepatitis infection, and mass screening of pregnant women for Hepatitis infections. The HBV vaccination provision should aim for universal coverage of under-five children.

## Data Availability

The original contributions presented in the study are included in the article/Supplementary Material, further inquiries can be directed to the corresponding author.
